# Exploring the role of gut microbiota in coronary atherosclerosis through lipoprotein-mediated cholesterol transport and distribution: A Mendelian randomization analysis

**DOI:** 10.1097/MD.0000000000050447

**Published:** 2026-09-04

**Authors:** Xiaobo Liu, Kailin Shen, Fangtao Zhu, Cunwei Cheng, Haibin Yu

**Affiliations:** a Department of Intervention, The Second Affiliated Hospital of Zhengzhou University, Zhengzhou, Henan, P. R. China.

**Keywords:** cholesterol, coronary atherosclerosis, gut microbiota, mediation analysis, Mendelian randomization analysis

## Abstract

We employed Mendelian randomization (MR) to explore causal relationships between gut microbiota (GM), coronary atherosclerotic heart disease (CAHD), and potential metabolic mediators. We utilized summary statistics from genome-wide association studies (GWAS), encompassing data on 473 GM traits from comprehensive microbiome GWAS, 61 lipoprotein-mediated cholesterol transport and distribution data from large-scale metabolic biomarker studies, and coronary atherosclerosis (CA) data from the GWAS catalog (study accession GCST90043957) involving 456,348 European participants. Bidirectional MR analyses were conducted to investigate the causal relationships between GM and CA. Two-sample Mendelian randomization analyses were performed to identify potential mediating metabolites and quantify the mediation proportion. Ultimately, the GM GCA-900066755, identified through MR as having a potential causal relationship, was selected to investigate its potential effects on CA by influencing cholesterol transport and distribution. Our results indicated that GCA-900066755 was positively associated with an increased risk of CA (odds ratio = 1.156). CA did not significantly affect the levels of GCA-900066755 (odds ratio = 1.009). GCA-900066755 was negatively correlated with total cholesterol levels in medium high-density lipoprotein, which reduced CA risk, and was positively correlated with total cholesterol levels in low-density lipoprotein (LDL), large LDL, medium LDL, and small LDL, which were positively associated with CA. Mediation analysis showed 7 data points mediating the association between GCA-900066755 and CA. Our MR study supports a causal relationship between specific GM groups and the risk of CAHD, highlighting that cholesterol traits are not merely outcomes associated with the relationship between GM and CAHD, but are important mediating factors. Understanding the biological mechanisms of these traits can provide a concrete foundation for future targeted interventions.

## 1. Introduction

Coronary atherosclerotic heart disease (CAHD) is a serious, progressive condition marked by the buildup of lipids and inflammatory cells within arterial walls.^[1–3]^ The intricate interplay between lipoproteins, cholesterol metabolism, and immune responses significantly contributes to the development of CAHD. Particularly, the accumulation and modification of low-density lipoprotein (LDL) leads to cholesterol deposits, known as plaques, forming in arteries. These plaques are absorbed by macrophages, creating “foam cells.”^[4–7]^

The gut microbiota (GM), a dynamic community of microorganisms residing in the gastrointestinal tract, influences various metabolic processes.^[8]^ Observational studies link GM to many cardiometabolic risk factors and diseases.^[9–11]^ The regulatory functions of the GM likely stem from metabolic products entering peripheral circulation either via the portal vein or directly through mesenteric absorption, acting as substrates or signaling molecules that influence host metabolism and health.^[12,13]^

Changes in the composition and function of the GM can significantly impact lipid metabolism and the levels of circulating lipoproteins.^[12,14–16]^ For example, Lei et al^[16]^ observed that short-chain fatty acids produced from dietary fiber fermentation by gut bacteria protect against dyslipidemia and metabolic syndrome. Specific gut bacteria also influence bile acid metabolism, impacting cholesterol homeostasis and lipid absorption and highlighting the critical role of the microbiota in the development of CAHD.^[15]^

Beyond metabolic functions, the GM also regulates immune responses, which are essential in the pathogenesis of CAHD.^[14,17–19]^ Gut-derived lipopolysaccharides can induce systemic inflammation, leading to endothelial dysfunction and the formation of atherosclerotic plaques.^[14]^ Additionally, alterations in GM composition have been linked to changes in immune cell populations and inflammatory markers, further establishing the connection between gut and heart health.^[19,20]^

Cholesterol metabolism is a dominant feature in CAHD, with triglyceride-rich lipoproteins and remnant cholesterol recognized as significant contributors to atherosclerotic cardiovascular disease. Elevated remnant cholesterol levels increase cardiovascular event risks, notwithstanding controlled LDL levels.^[7]^ Therefore, examining the relationship between GM, cholesterol metabolism, and CAHD may offer new treatment and prevention strategies for CAHD.

However, owing to the unique characteristics of the GM, most studies on this topic lack sufficient reproducibility.^[21–23]^ Factors such as obesity, medication, diet, and alcohol consumption are significant confounders of gut microbial composition. These studies often encounter biases from reverse causation and confounding, complicating causal relationship assessments.^[22]^ Therefore, innovative methods like Mendelian randomization (MR) can provide more reliable results in GM studies.

We explored the role of GM in CAHD through MR analysis, focusing on the mediating effects of lipoprotein-mediated cholesterol transport and distribution. By using mediation analysis, we aim to understand the complex interactions between GM and cardiovascular health and identify new therapeutic strategies to reduce the global burden of cardiovascular diseases.

## 2. Materials and methods

### 2.1. Research design

We utilized the 2-sample Mendelian randomization (TSMR) method to investigate the bidirectional causal relationship between GM and CAHD. TSMR is a statistical method that leverages genetic variants as instrumental variables, allowing us to infer causality without the influence of confounding factors.

**Figure 1.**
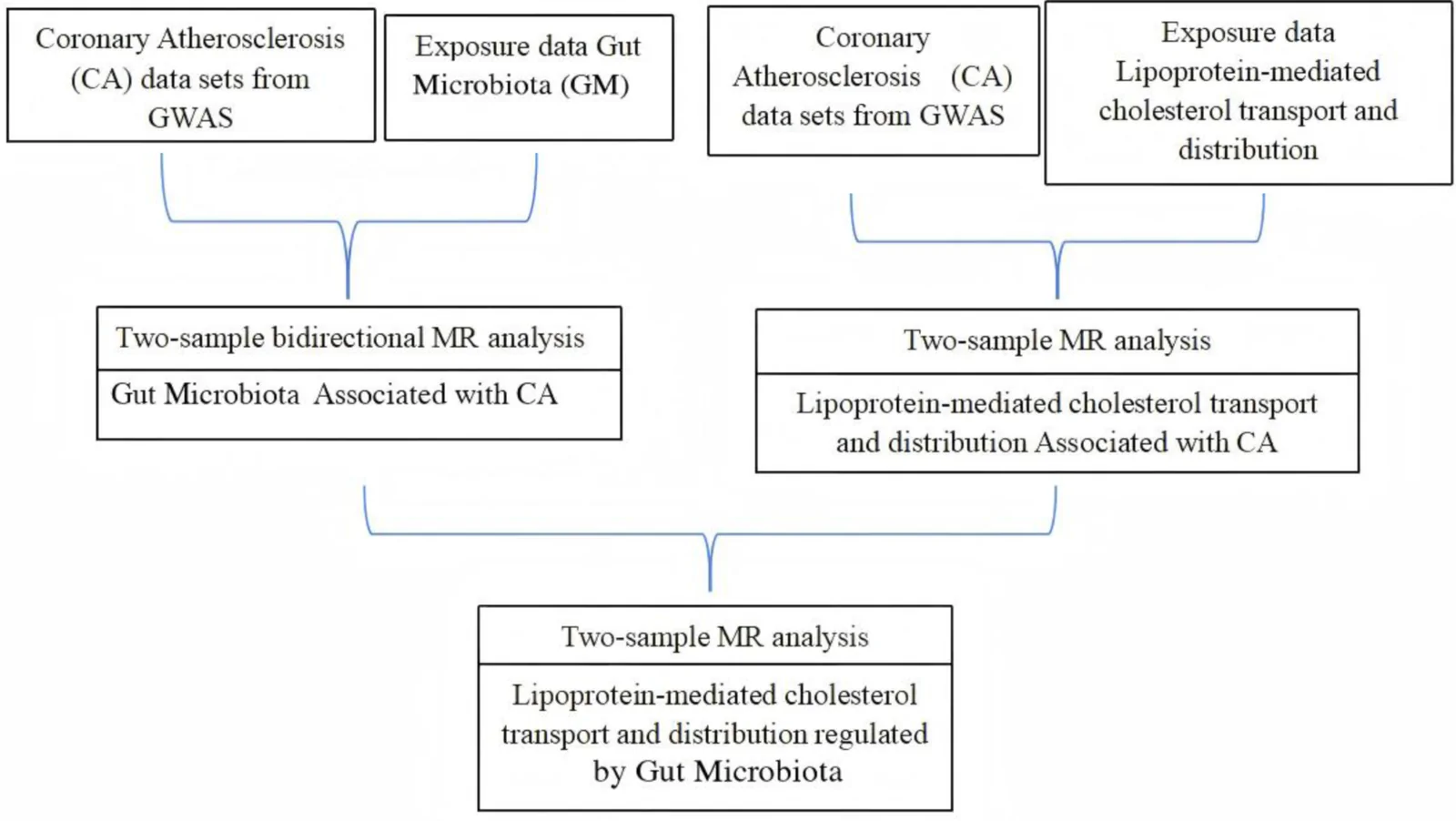
Flowchart detailing the Mendelian randomization (MR) study design. GWAS = genome-wide association study.

In this study, we 1st conducted a 2-sample MR analysis to explore the bidirectional causal link between GM and CAHD, assessing whether GM is associated with the risk of CAHD. Next, we performed an MR analysis on lipoprotein-mediated cholesterol transport and distribution to evaluate its relationship with CAHD, aiming to understand the potential role of cholesterol metabolism in the association between GM and CAHD. Finally, we employed a 2-step MR design to investigate whether cholesterol transport and distribution act as mediators in the causal pathway between GM and CAHD. This analysis seeks to clarify how cholesterol metabolism mediates the effect of GM on CAHD risk. Figure 1 illustrates the full study design.

### 2.2. Data sources

The genome-wide association study (GWAS) data for GM were sourced from the FINRISK 2002 cohort, comprising 5959 individuals of European descent.^[24]^ Cholesterol-related metabolic information was derived from a comprehensive analysis of 233 serum or plasma metabolic traits quantified by nuclear magnetic resonance metabolomics in 136,016 predominantly European individuals.^[25]^ Coronary atherosclerosis (CA) data were obtained from the GWAS Catalog, with the GCST ID GCST90043957, including 16,041 European ancestry cases and 440,307 European ancestry controls.^[26]^ All data were publicly available, thus not requiring ethical approval.

### 2.3. Selection and validation of instrumental variable single nucleotide polymorphisms (SNPs)

We used 3 criteria to select SNPs. First, SNPs that reached genome-wide significance levels (*P* < 5 × 10^−5^) were chosen, thus supporting the association assumption of MR. Second, linkage disequilibrium analysis ensured the independence of these SNPs. SNPs in linkage disequilibrium and close to other SNPs with higher *P* values were excluded; particularly, SNPs within a 10,000 kb range of the most significant SNP with an *r*^2^ > 0.001 were removed. Finally, to control for potential bias, we calculated *F* statistics to evaluate our selected instrumental variables for weak instrument bias. An *F* statistic >10 indicates no weak instrument bias, thus further validating the association assumption. The formula for the *F* statistic is *F* = [(*N* − *K* − 1)/*K*] × [*R*^2^/(1 − *R*^2^)], where *N* is the sample size for the exposure factor, *K* is the number of instrumental variables, and *R*^2^ is the proportion of variance in the exposure factor explained by the instrumental variables.^[27,28]^

### 2.4. MR analysis design

**Figure 2.**
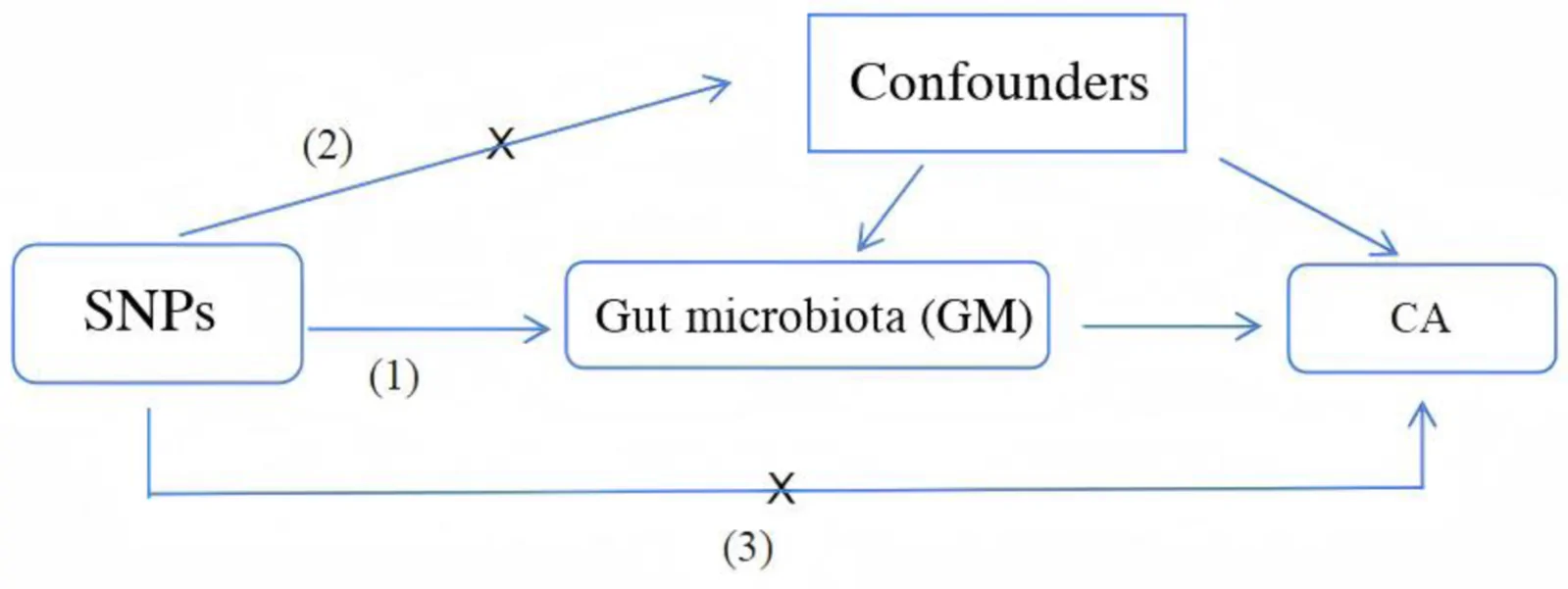
The 3 key assumptions of MR research are: (1) a single nucleotide polymorphism (SNP) is closely related to various lipid levels; (2) the SNP is independent of other known confounding factors; (3) the SNP only affects the risk of CAs through various lipids. *X* indicates that the SNP chosen as an instrumental variable is not directly related to confounding factors and outcomes. CA = coronary atherosclerosis, MR = Mendelian randomization

MR methods must satisfy 3 core assumptions: association, independence, and exclusion restriction (Fig. 2). First, association: the instrumental variable must be strongly associated with the exposure factor. Second, independence: the instrumental variable should not be associated with any confounders related to the “exposure-outcome” relationship. Third, exclusion restriction: the instrumental variable can influence the outcome variable only through the exposure factor, without any alternative pathways. These assumptions are critical to ensuring the validity of the MR analysis and the reliability of its causal inferences. In our analysis, SNPs were used as instrumental variables.^[28–30]^

TSMR analysis was conducted using random-effects inverse-variance weighted (IVW), weighted median, and MR-Egger methods to assess the potential causal relationship between cholesterol metabolism and CAHD.^[31]^ Traditional IVW analysis may be prone to bias from invalid instruments or pleiotropy. Therefore, we included sensitivity analyses to test the validity and robustness of the IVW results. The average level of pleiotropy bias was assessed through MR-Egger regression, with a *P* value <.05 indicating pleiotropy between the exposure and outcome variables. MR pleiotropy residual sum and outlier (MR-PRESSO) was utilized to detect horizontal pleiotropy. For heterogeneity testing, Cochran *Q* method was employed, with a *P* value <.05 indicating the presence of heterogeneity among the included instrumental variable SNPs, suggesting that the study results are influenced by heterogeneity. The “leave-one-out” method involved sequentially removing each SNP and conducting MR analysis with the remaining SNPs to observe changes in the overall effect estimate. If the removal of any single SNP resulted in a significant change in the effect estimate, it may indicate that the SNP negatively affects the overall estimate.^[31,32]^

All statistical analyses were performed using the “TwoSampleMR” package in R software, version 4.3.2.

## 3. Results

### 3.1. Association between gut microbiota and CAHD through 2-sample bidirectional MR analysis

The analysis identified 19 bacterial traits associated with CAHD risk. Protective factors included Desulfovibrionaceae (odds ratio [OR] = 0.8909, 95% confidence interval [CI]: 0.8246–0.9625, *P* = .0034), Desulfovibrionales (OR = 0.8752, 95% CI: 0.8077–0.9483, *P* = .0011), Marinilabiliaceae (OR = 0.7056, 95% CI: 0.5395–0.9228, *P* = .0108), CAG-194 sp002441865 (OR = 0.8393, 95% CI: 0.7080–0.9950, *P* = .0436), *Bifidobacterium angulatum* (OR = 0.9249, 95% CI: 0.8570–0.9980, *P* = .0447), *Blautia A* sp000285855 (OR = 0.7396, 95% CI: 0.5862–0.9332, *P* = .0110), Faecalibacterium sp002160895 (OR = 0.8070, 95% CI: 0.6543–0.9954, *P* = .0451), DTU024 sp002411105 (OR = 0.8095, 95% CI: 0.6569–0.9975, *P* = .0473), GCA-900066495 (OR = 0.8934, 95% CI: 0.7984–0.9996, *P* = .0492), *Johnsonella ignava* (OR = 0.7910, 95% CI: 0.6372–0.9821, *P* = .0337), and UBA1206 sp000433115 (OR = 0.8996, 95% CI: 0.8164–0.9912, *P* = .0325). Risk factors included CAG-776 sp000438195 (OR = 1.1135, 95% CI: 1.0043–1.2346, *P* = .0412), *Olsenella C* (OR = 1.3294, 95% CI: 1.0847–1.6293, *P* = .0061), UCG-010 sp003150215 (OR = 1.1080, 95% CI: 1.0092–1.2164, *P* = .0313), Fibrobacterales (OR = 1.7628, 95% CI: 1.0972–2.8323, *P* = .0191), Eisenbergiella sp900066775 (OR = 1.1214, 95% CI: 1.0243–1.2276, *P* = .0131), *Enorma massiliensis* (OR = 1.1030, 95% CI: 1.0164–1.1970, *P* = .0188), GCA-900066755 sp900066755 (OR = 1.2309, 95% CI: 1.0731–1.4120, *P* = .0030), and GCA-900066755 (OR = 1.1558, 95% CI: 1.0333–1.2928, *P* = .0113).

The results of pleiotropy and heterogeneity analyses showed that CAG-776 sp000438195 had significant pleiotropy and heterogeneity (heterogeneity *P* value = .011, MR-PRESSO = 0.015). UBA1206 sp000433115 had significant pleiotropy (MR-PRESSO = 0.028). Other traits did not show significant heterogeneity or pleiotropy. Among these, excluding CAG-776 sp000438195 and UBA1206 sp000433115, the *P* values for CAG-194 sp002441865, *Bifidobacterium angulatum*, Faecalibacterium sp002160895, DTU024 sp002411105, GCA-900066495, *Johnsonella ignava*, and UCG-010 sp003150215 were approximately 0.05. These results are statistically significant; however, their persuasive power is relatively weak and thus they are not included in the study. Table 1 presents the MR results of GM and CA risk.

**Table 1:** Associations between the genetically predicted gut microbiota and the risk of coronary atherosclerosis.

	SNPs (n)		MR-Egger	Weighted median	Inverse-variance weighted
Desulfovibrionaceae	19	OR (95% CI)	1.003 (0.693–1.052)	0.913 (0.819–1.017)	0.891 (0.825–0.962)
		*P* val	.157	.099	.003
Desulfovibrionales	21	OR (95% CI)	0.862 (0.686–1.082)	0.902 (0.804–1.013)	0.875 (0.808–0.948)
		*P* val	.215	.082	.001
Marinilabiliaceae	16	OR (95% CI)	0.473 (0.251–0.893)	0.707 (0.478–1.045)	0.706 (0.540–0.923)
		*P* val	.036	.082	.011
Blautia A sp000285855	12	OR (95% CI)	0.585 (0.332–1.028)	0.793 (0.601–1.047)	0.740 (0.586–0.933)
		*P* val	.092	.101	.011
Johnsonella ignava	13	OR (95% CI)	0.685 (0.403–1.165)	0.757 (0.567–1.010)	0.791 (0.637–0.982)
		*P* val	.190	.058	.034
Olsenella C	14	OR (95% CI)	1.204 (0.775–1.869)	1.321 (1.051–1.659)	1.329 (1.085–1.629)
		*P* val	.426	.017	.006
Fibrobacterales	14	OR (95% CI)	0.737 (0.269–2.014)	1.384 (0.718–2.666)	1.763 (1.097–2.832)
		*P* val	.563	.332	.019
Eisenbergiella sp900066775	15	OR (95% CI)	1.174 (0.977–1.411)	1.105 (0.973–1.254)	1.121 (1.024–1.228)
		*P* val	.110	.124	.013
Enorma massiliensis	30	OR (95% CI)	1.115 (0.974–1.276)	1.104 (0.981–1.241)	1.103 (1.016–1.197)
		*P* val	.126	.100	.019
GCA-900066755 sp900066755	19	OR (95% CI)	1.219 (0.901–1.650)	1.240 (1.009–1.524)	1.231 (1.073–1.412)
		*P* val	.216	.041	.003
GCA-900066755	25	OR (95% CI)	1.095(0.847–1.416)	1.201(1.025–1.408)	1.156(1.033–1.293)
		*P* val	.494	.023	.011

CI = confidence interval, MR = Mendelian randomization, OR = odds ratio, SNPs = Single nucleotide polymorphisms.

### 3.2. Exploring the relationships between GCA-900066755 and cholesterol metabolism in CAHD

**Figure 3.**
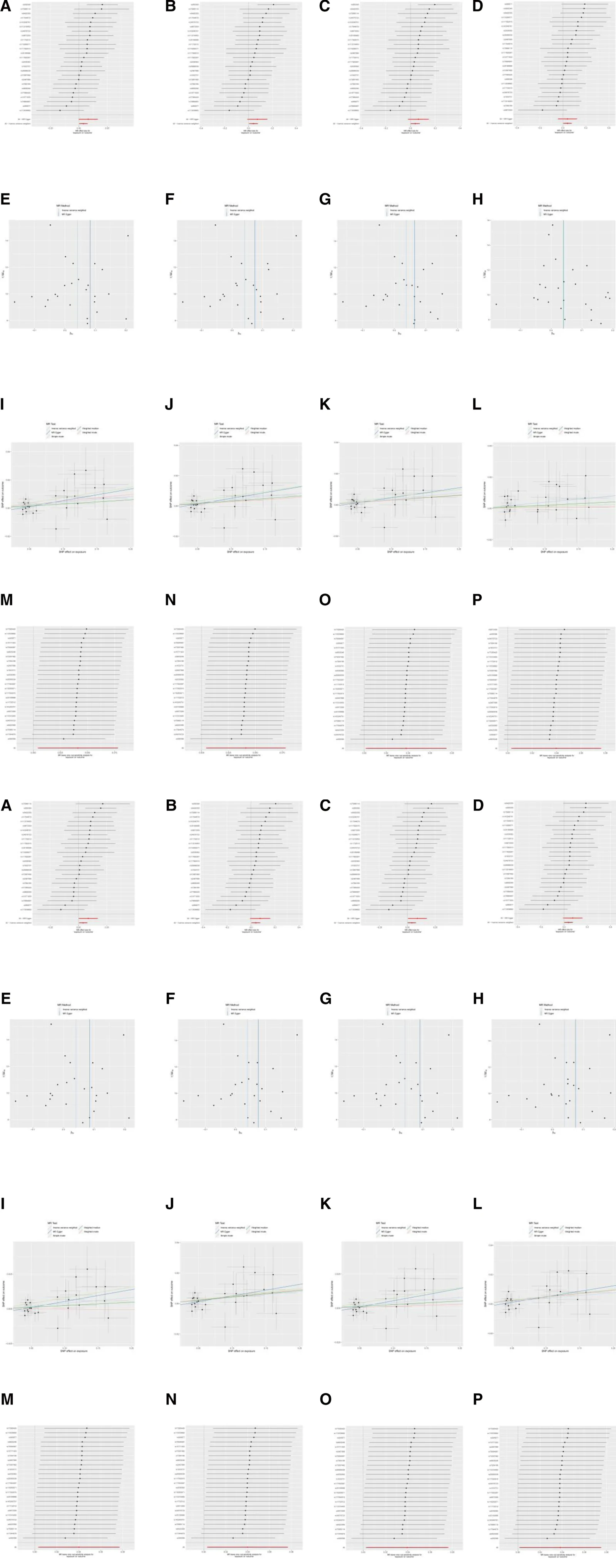
Causal effects of gut microbiota on lipoprotein-mediated cholesterol transport and distribution 1 (A–D) forest plots, 1 (E–H) funnel plots, and 1 (I–L) scatter plots of GCA-900066755 on free cholesterol in large low-density lipoprotein (LDL), free cholesterol in medium LDL, free cholesterol in small LDL, and total cholesterol in large LDL 1 (M–P) leave-one-out sensitivity analysis of single nucleotide polymorphisms (SNPs) associated with GCA-900066755 and free cholesterol in large LDL, free cholesterol in medium LDL, free cholesterol in small LDL, and total cholesterol in large LDL. 2 (A–D) forest plots, 2 (E–H) funnel plots, and 2 (I–L) scatter plots of GCA-900066755 on total cholesterol in medium LDL, total cholesterol in small LDL, total cholesterol levels in LDL, and total cholesterol levels in medium high-density lipoprotein (HDL) 2 (M–P) leave-one-out sensitivity analysis of SNPs associated with GCA-900066755 and total cholesterol in medium LDL, total cholesterol in small LDL, total cholesterol levels in LDL, and total cholesterol levels in medium HDL.

We identified cholesterol transport and distribution data associated with CAHD and, through MR analysis, screened 51 positive data sets out of 61. Extensive MR analyses were conducted on the remaining 10 GM traits with the screened positive data to explore their complex relationships with cholesterol transport and distribution traits. Ultimately, we observed that only GCA-900066755 had a potential causal relationship with cholesterol transport and distribution. Additionally, a reverse MR analysis was conducted, showing that GCA-900066755 did not exhibit significant changes caused by CAHD (*P* = .3519, β = 0.0091). No further evidence supported a causal effect of CAHD on the other taxa identified in the aforementioned results. The MR results showed that at the genetic level, GCA-900066755 regulated 8 cholesterol metabolic traits, decreasing the expression of 1 trait and increasing the expression of the remaining 7 traits. GCA-900066755 is negatively correlated with total cholesterol levels in medium high-density lipoprotein (HDL), which reduces the risk of CAHD. Conversely, it is positively correlated with total cholesterol levels in LDL, large LDL, free cholesterol in large LDL, medium LDL, free cholesterol in medium LDL, small LDL, and free cholesterol in small LDL, which are positively associated with an increased risk of CAHD. These results suggest that the increased risk of CAHD caused by elevated levels of GCA-900066755 may be partially due to cholesterol-related metabolism. Figures S1 and S2, Supplemental Digital Contents 1 and 2 show the causal effects of lipoprotein-mediated cholesterol transport and distribution on CAHD. Table 2 and Figure 3 present the associations between the genetically predicted GM and lipoprotein-mediated cholesterol transport and distribution.

**Table 2:** Associations between the genetically predicted gut microbiota and lipoprotein-mediated cholesterol transport and distribution.

		MR-Egger	Weighted median	Inverse-variance weighted
Free cholesterol in large LDL	OR (95% CI)	1.066 (0.980–1.159)	1.031 (0.978–1.087)	1.039 (1.001–1.078)
	*P* val	.149	.258	.042
Free cholesterol in medium LDL	OR (95% CI)	1.079 (0.992–1.173)	1.044 (0.992–1.099)	1.041 (1.003–1.080)
	*P* val	.089	.100	.034
Free cholesterol in small LDL	OR (95% CI)	1.077 (0.991–1.171)	1.048 (0.992–1.107)	1.039 (1.001–1.078)
	*P* val	.094	.095	.042
Total cholesterol in large LDL	OR (95% CI)	1.077 (0.991–1.171)	1.033 (0.981–1.089)	1.042 (1.004–1.081)
	*P* val	.095	.220	.029
Total cholesterol in medium LDL	OR (95% CI)	1.089 (1.002–1.185)	1.024 (0.970–1.081)	1.041 (1.003–1.080)
	*P* val	.058	.390	.034
Total cholesterol in small LDL	OR (95% CI)	1.094 (1.006–1.190)	1.027 (0.972–1.084)	1.040 (1.002–1.079)
	*P* val	.047	.343	.037
Total cholesterol levels in LDL	OR (95% CI)	1.086 (0.999–1.181)	1.030 (0.978–1.084)	1.042 (1.004–1.081)
	*P* val	.066	.262	.029
Total cholesterol levels in medium HDL	OR (95% CI)	1.041 (0.958–1.131)	1.021 (0.968–1.077)	1.040 (1.003–1.080)
	*P* val	.356	.451	.035

CI = confidence interval, HDL = high-density lipoprotein, LDL = low-density lipoprotein, MR = Mendelian randomization, OR = odds ratio, SNPs = Single nucleotide polymorphisms.

### 3.3. Mediation analysis of gut microbiota, cholesterol-related metabolism, and CAHD

To investigate the mediating effects of the 8 cholesterol-related metabolic traits, we calculated their indirect effects and proportions. The overall effect size was represented by the β value between GCA-900066755 and CAHD, determined through TSMR. The indirect effect was derived by multiplying the β value of GCA-900066755 on the cholesterol-related traits by the β value of the cholesterol-related traits on CAHD. The direct effect was calculated by subtracting the indirect effect from the overall effect. The mediation effects were as follows: total cholesterol levels in medium HDL showed a mediation effect of −0.0138 with a mediation proportion of 9.54%; total cholesterol levels in LDL had a mediation effect of 0.0177 with a mediation proportion of 12.25%; total cholesterol in large LDL exhibited a mediation effect of 0.0173 with a mediation proportion of 11.92%; free cholesterol in large LDL showed a mediation effect of 0.0160 with a mediation proportion of 11.04%; total cholesterol in medium LDL had a mediation effect of 0.0179 with a mediation proportion of 12.36%; free cholesterol in medium LDL exhibited a mediation effect of 0.0175 with a mediation proportion of 12.08%; total cholesterol in small LDL showed a mediation effect of 0.0169 with a mediation proportion of 11.66%; and free cholesterol in small LDL had a mediation effect of 0.0166 with a mediation proportion of 11.45%. These calculations illustrate the extent to which the cholesterol-related traits mediate the relationship between GCA-900066755 and CAHD. Table 3 presents the mediation analysis results.

**Table 3:** Mediation analysis results of gut microbiota GCA-900066755, lipoprotein-mediated cholesterol transport and distribution, and coronary atherosclerosis.

Trait	Beta1	Beta2	Beta12	Beta12_p
Free cholesterol in large LDL	0.0382	0.4182	0.0160	11.04%
Free cholesterol in medium LDL	0.0399	0.4383	0.0175	12.08%
Free cholesterol in small LDL	0.0383	0.4331	0.0166	11.45%
Total cholesterol in large LDL	0.0412	0.4193	0.0173	11.92%
Total cholesterol in medium LDL	0.0402	0.4454	0.0179	12.36%
Total cholesterol in small LDL	0.0395	0.4276	0.0169	11.66%
Total cholesterol levels in LDL	0.0412	0.4305	0.0177	12.25%
Total cholesterol levels in medium HDL	0.0396	−0.3486	−0.0138	9.54%

Beta1 = the total effect of gut microbiota GCA-900066755 on coronary atherosclerosis, Beta12 = mediating effect, Beta12_p = proportion of mediation effect, Beta2 = the effect of lipoprotein-mediated cholesterol transport and distribution on coronary atherosclerosis, HDL = high-density lipoprotein, LDL = low-density lipoprotein.

## 4. Discussion

In this MR study, we examined the causal relationships between GM, CAHD, and potential metabolic mediators. Our findings provide new insights into how specific GM taxa contribute to CAHD and the mediating effects of cholesterol-related metabolic traits.

In recent years, studies on the relationship between GM-derived metabolites and CA have increased.^[33–35]^ Previous studies have shown a significant correlation between CA and GM. For example, Sergi et al^[35]^ observed that CACS-associated bacterial species are linked to at least 1 plasma metabolite and tend to cluster into different groups based on their association with the metabolome. Akihiro et al^[33]^ explored how GM influences the characteristics of coronary artery plaques, focusing on potential mechanisms that promote plaque development and instability. However, these studies are primarily focused on inflammation. Our study, in contrast, delves into the lipid metabolism-related pathways and their mediating effects on CAHD. This approach provides a new perspective on how GM may influence cardiovascular health via cholesterol metabolism and offers potential therapeutic targets.

Our MR analysis identified several bacterial traits significantly associated with CAHD risk, with GCA-900066755 being a notable risk factor. The positive association between GCA-900066755 and CAHD underscores the significant role of GM in cardiovascular health. This is consistent with previous studies indicating that changes in GM composition can influence lipid metabolism and cardiovascular disease risk.^[9–11]^ Lei et al^[16]^ discovered that dietary fiber fermentation by gut bacteria produces short-chain fatty acids that protect against dyslipidemia and metabolic syndrome. Regarding cholesterol metabolism, our MR analysis revealed that GCA-900066755 is negatively correlated with total cholesterol levels in medium HDL, reducing CAHD risk. Conversely, it is positively correlated with total cholesterol levels in LDL, large LDL, medium LDL, and small LDL, which are associated with increased CAHD risk. This highlights the complex relationship between GM and cholesterol transport and distribution, explaining the observed associations. Previous studies have also noted that certain gut bacteria can affect bile acid metabolism, which influences cholesterol homeostasis and lipid absorption.^[15]^ The mediation analysis quantified the indirect effects of cholesterol-related metabolic traits on the relationship between GCA-900066755 and CAHD. The mediation proportions ranged from 9.54% to 12.36%, with total cholesterol levels in medium LDL showing the highest mediation proportion. These findings suggest that the impact of GCA-900066755 on CAHD is partly mediated through its effect on cholesterol metabolism, highlighting the importance of lipid-related pathways in the gut-heart axis.^[36]^

Importantly, lipid metabolism represents a clinically modifiable pathway. Lifestyle factors such as dietary patterns and physical activity are known to influence both GM composition and circulating lipoprotein profiles.^[12,16]^ In the LIGHT trial and its sub-studies, mobile application–supported lifestyle interventions significantly reduced ASCVD risk scores and improved cardiopulmonary fitness and autonomic function in high-risk individuals.^[37–39]^ Although these studies did not directly assess GM, their observed improvements in lipid parameters and cardiovascular risk markers are consistent with the microbiota–lipid mediation pathway suggested by our MR findings. Furthermore, nutritional status itself has been identified as an independent determinant of cardiovascular outcomes in elderly cardiac populations, underscoring the importance of integrating dietary optimization into preventive strategies.^[40]^

However, our analysis does not align with existing experimental studies and, in some aspects, even contradicts them. There is limited research on GCA-900066755, but current experimental results tend to suggest that this bacterium may be beneficial to human health. In our MR analysis, GCA-900066755 was found to be positively associated with CAHD risk, yet other studies have linked reduced abundance of GCA-900066755 to the onset of certain diseases.^[41]^ For instance, in gastrointestinal microbiome studies of patients with gallstone disease, the abundance of GCA-900066755 was significantly decreased. Other studies have shown that fucoidan sulfates in seaweed possess lipid-lowering effects and can increase the abundance of beneficial bacteria, including Ruminococcaceae_NK4A214_group, GCA-900066755, and Eubacterium. These bacteria are positively correlated with fecal DCA, β-MCA, and HDCA, and are able to regulate lipid metabolism in diet-induced hyperlipidemic mice. This evidence suggests that GCA-900066755 may have potential probiotic effects. Although our analysis indicates a positive association between GCA-900066755 and an increased risk of CAHD, experimental data suggest that this bacterium may be beneficial in certain contexts. Therefore, further research is needed to elucidate its specific mechanisms of action under different health conditions to better understand the complex effects of GM on cardiovascular health.

We employed a robust 2-sample MR design, utilizing large-scale GWAS data to explore the causal relationships between GM, cholesterol metabolism, and CAHD. The application of MR-PRESSO and Cochran *Q* test ensured the reliability of our results by accounting for potential pleiotropy and heterogeneity. Additionally, the bidirectional MR approach allowed us to assess reverse causation, confirming that CAHD does not significantly influence the levels of GCA-900066755.^[31,32]^ This approach enhances the credibility of our findings and addresses the limitations of previous studies that focused only on unidirectional analyses.

Our study enhances previous research by further elucidating the role of GM in lipid metabolism and cardiovascular disease.^[42]^ For example, a study by Bäckhed et al^[43]^ demonstrated that GM can regulate host metabolism by modulating bile acid profiles and interacting with the host’s immune system, which is critical in the pathogenesis of atherosclerosis. Similarly, Liu et al^[20]^ observed that the GM of patients with coronary artery disease influenced bile acid metabolism, leading to vascular dysfunction in mice. Our findings are also consistent with those of Tuomisto et al,^[11]^ who demonstrated that gut microbial composition is associated with coronary artery disease through mechanisms involving lipid metabolism and inflammation.

However, our study has certain limitations. First, the accuracy of MR analysis heavily depends on the validity of the SNPs used as instrumental variables. These SNPs must be strongly associated with the exposure and should influence the outcome variable only through the exposure. If other pathways affect the outcome, such as horizontal pleiotropy, bias may be introduced. Second, our analysis was restricted to populations of European descent, which may limit the generalizability of our findings to other ethnic groups due to genetic diversity and differing environmental exposures. Population-specific genetic variations can influence allele frequencies and linkage disequilibrium patterns, potentially affecting the transferability of our results to non-European populations. Therefore, caution should be exercised when extrapolating these findings to other ethnic groups. Additionally, our study relied on summary data for CAHD cases, preventing subgroup analyses, especially those stratified by sex and age. This limitation reduced our ability to understand disease dynamics across different demographic groups. The quality and accuracy of MR analysis also depend on the integrity and availability of the underlying GWAS data. Any errors or deficiencies in the data could affect the results of the MR analysis. Despite MR analysis indicating a potential causal relationship, identifying the specific biological mechanisms remains a challenge. Furthermore, MR analysis cannot provide information on the size of the intervention effect.^[28]^ While this study offers valuable insights, these limitations should be considered when interpreting the results and generalizing the findings. Future research should involve diverse populations, including non-European ethnic groups, to validate our findings and enhance their generalizability. Such studies would help determine whether the observed associations are consistent across different genetic backgrounds and environmental contexts. Experimental studies are also necessary to investigate the functional roles of specific GM in cholesterol metabolism and cardiovascular health.

## 5. Conclusion

In summary, our MR study supports a causal relationship between specific GM and the risk of CAHD, with cholesterol-related metabolic traits as mediators. Our findings highlight the potential of targeting GM and cholesterol metabolism as novel approaches to mitigate the burden of CAHD. Further research is warranted to elucidate the underlying mechanisms and develop effective microbiome-based interventions.

## Author contributions

**Conceptualization:** Kailin Shen, Haibin Yu.

**Data curation:** Xiaobo Liu, Kailin Shen, Fangtao Zhu, Haibin Yu.

**Formal analysis:** Xiaobo Liu, Kailin Shen, Haibin Yu.

**Funding acquisition:** Haibin Yu.

**Investigation:** Xiaobo Liu, Kailin Shen, Haibin Yu.

**Methodology:** Xiaobo Liu, Kailin Shen, Haibin Yu.

**Project administration:** Xiaobo Liu, Kailin Shen, Fangtao Zhu, Haibin Yu.

**Resources:** Xiaobo Liu, Kailin Shen, Haibin Yu.

**Software:** Xiaobo Liu, Kailin Shen, Haibin Yu.

**Supervision:** Fangtao Zhu, Cunwei Cheng, Haibin Yu.

**Validation:** Xiaobo Liu, Kailin Shen, Haibin Yu.

**Visualization:** Xiaobo Liu, Kailin Shen, Haibin Yu.

**Writing – original draft:** Xiaobo Liu, Kailin Shen, Fangtao Zhu, Cunwei Cheng.

**Writing – review & editing:** Xiaobo Liu, Fangtao Zhu, Cunwei Cheng, Haibin Yu.




